# Ticks on migrating birds in southwestern Poland: occurrence of *Ixodes ricinus* and the first Polish record of *Haemaphysalis concinna* on birds

**DOI:** 10.1007/s10493-025-01070-2

**Published:** 2025-10-20

**Authors:** Dagmara Dyczko, Lucyna Hałupka, Beata Czyż, Aleksandra Czułowska, Dorota Kiewra

**Affiliations:** 1 Department of Microbial Ecology and Acaroentomology, Faculty of Biological Sciences, University of Wrocław, Przybyszewskiego 63/77, Wrocław, 51- 148 Poland; 2 Department of Avian Ecology, Faculty of Biological Sciences, University of Wrocław, Sienkiewicza 21, Wrocław, 50-335 Poland

**Keywords:** Acrocephalus arundinaceus, Great reed warbler, Migratory birds, Haempahysalis concinna, Ticks, Poland

## Abstract

During ornithological research conducted at the Milicz Fishponds Nature Reserve (Barycz Valley Landscape Park, southwestern Poland) from May to July 2024, a total of 245 birds were captured. Four ticks were collected: two *Ixodes ricinus* larvae and two *Haemaphysalis concinna* nymphs. We present the first confirmed record of *H*. *concinna* parasitising a great reed warbler (*Acrocephalus arundinaceus*) in Poland. Identification of *H*. *concinna* was based on both morphological identification keys and molecular analysis of the *COI* gene. This finding expands current knowledge on the biodiversity of ticks parasitising birds in Poland and highlights the need for further research on the role of migratory birds in the dispersal of ticks across Central Europe.

## Introduction

The role of birds, particularly migratory species, in the dispersal of ticks and their associated pathogens is increasingly recognized as a significant factor in the ecology of tick-borne diseases. Notably, migratory birds can act as transporters for ticks and carriers for tick-borne pathogens, moving these ectoparasites over vast geographical expanses (Choi et al. 2014; Flaisz et al. 2017; Buczek et al. 2020). A number of studies in Europe and Asia have shown that migratory passerines can transport ticks over considerable distances, facilitating the spread of these arthropods to new geographic regions (Hornok et al. 2016). In migratory bird systems such as those in North America and Europe, extensive research has shown that birds play a key role in shaping tick and tick-borne pathogen dynamics by introducing ticks into new regions and supporting their spread across ecological barriers. For example, Ogden et al. (2008) demonstrated that migratory birds were instrumental in the northward expansion of *Ixodes scapularis* in Canada and the concurrent introduction of *Borrelia burgdorferi* and *Anaplasma phagocytophilum*. Similarly, Cohen et al. (2015) showed that Neotropical migratory birds arriving in the United States carry non-native tick species, facilitating the invasion of both ticks and associated pathogens into new environments. Hamer et al. (2012) further highlighted that in urban environments like Chicago, wild birds support tick populations and pathogen circulation, indicating that bird movement also plays a role in sustaining local transmission cycles. In Europe, Klitgaard et al. (2019) found that migratory birds during spring and autumn in Denmark carried *Ixodes ricinus* infected with multiple pathogens, underlining their role in transregional dissemination of tick-borne diseases across the continent. This is particularly critical in Central Europe, where such birds migrate from warmer climates, bringing with them various ectoparasites (Hornok et al. 2016; Flaisz et al. 2017; Liu et al. 2024). Observations from Hungary suggest that the appearance of *Haemaphysalis concinna* in Europe, including Poland, may be related to the migration of birds from Siberia and the Far East (Flaisz et al. 2017). Furthermore, research conducted in Korea indicates that *H. concinna* ticks may be appearing in new locations due to changing bird migration routes and climate change (Choi et al. 2014).

*Haemaphysalis concinna* is widely distributed in the Eurasian continent, predominantly in China, Russia, and Central Europe, including Germany, the Czech Republic, Slovakia, Hungary, and Austria (Estrada-Peña et al. 2017; Rubel et al. 2018; Liu et al. 2024). In Central Europe, it is the second most abundant tick species collected from birds, after *I. ricinus*, and the third most abundant tick species flagged from vegetation (Rubel et al. 2018). In Poland, however, *H. concinna* has rarely been observed thus far, with its occurrence being predominantly confined to the western and south-eastern regions of the country (Dwużnik et al. 2019; Kiewra et al. 2019; Zięba et al. 2019). Although *H. concinna* can parasitize over 100 host species, half of which are birds (Liu et al. 2024), it has never been found on birds in Poland in spite of dozens of research investigating ticks on birds (Siuda et al. 2006) and thousands of birds inspected for the presence of ticks in recent years (Ciebiera et al. 2019; Zając et al. 2022). Given that this tick species has been recorded on birds in neighboring countries (Hubálek et al. 1996; Flaisz et al. 2017; Keve et al. 2024), its apparent absence in Poland is likely due to the fact that previous studies may not have included areas where *H. concinna* was noted. As a result, key habitats where birds could acquire this tick may have been overlooked. Therefore, the lack of records from bird hosts in Poland is more plausibly attributed to gaps in geographic and ecological coverage rather than to the species’ true absence.

*Haemaphysalis concinna* has been observed to inhabit a wide range of environments, with a preference for humid and well-lit habitats such as reedbeds, riverbanks, lake shores, forest edges, and clearings. It is particularly associated with deciduous and mixed forests with dense undergrowth, but also occurs in forest steppe and wet steppe zones, suggesting considerable ecological flexibility (Rubel et al. 2018). The habitat use partly overlaps with the stopover and breeding sites of reed-associated migratory birds, making them likely hosts for this tick species. The nymphs and larvae of this thermophilic tick species demonstrate seasonal activity patterns that align closely with the migratory cycles of its avian hosts, enhancing their chances of successful dispersal and establishment in new areas (Keve et al. 2024; Liu et al. 2024).

*Haemaphysalis concinna* is known to transmit a range of pathogens of medical and veterinary importance, including *(A) phagocytophilum* (agent of human and animal granulocytic anaplasmosis), *(B) burgdorferi* sensu lato (causative agent of Lyme borreliosis), spotted fever group rickettsiae such as *Rickettsia helvetica* and *R. raoultii*, *Babesia microti* and a novel *Babesia* genotype related to *Babesia crassa* (agents of babesiosis), and viruses including the severe fever with thrombocytopenia syndrome virus (SFTSV) (Meng et al. 2015; Rubel et al. 2018; Dwużnik-Szarek et al. 2021; Liu et al. 2024). The association between *H. concinna* and birds has significant implications for public health, as birds can act not only as long-distance transporters of infected ticks, but may also serve as competent reservoir hosts for certain pathogens. For example, wild birds have been identified as reservoirs for *B. burgdorferi* s.l., contributing to local transmission cycles in addition to facilitating pathogen dispersal over large geographic areas (Humair 2002; Benskin et al. 2009; Hornok 2017). This paper documents for the first time the presence of *H. concinna* on birds in Poland.

## Materials and methods

### Fieldwork

Ornithological research at the Milicz Fishponds Nature Reserve (Barycz Valley Landscape Park, southwestern Poland) was conducted between 17 May and 31 July 2024 as a part of a long-term study on the breeding biology of Eurasian reed warblers (Orłowski et al. 2016; Wojczulanis-Jakubas et al. 2023; Hałupka et al. 2024). The Milicz Fishponds Nature Reserve is a group of fishponds located in the Barycz River valley. Due to their significance as a habitat and breeding ground for water birds, these ponds are designated as a nature reserve, forming part of the larger protected area known as the Barycz Valley Landscape Park (Evans 2009). Birds were caught every 1–4 days (during a total of 55 days) using ca. 15 mist-nets located on the study plot of 3 ha. The plot has a roughly rectangular shape (approximately 150 × 270 m; centre at 51.5385°N, 17.3390°E) and is situated in an extensive reedbed with a dominant common reed (*Phragmites australis*), accompanied by patches of bittersweets (*Solanum dulcamara*), cattails (*Typha angustifolia*) and other herbaceous vegetation.

After mist-netting each bird was identified to species level, aged and sexed (Svensson 1992), and subsequently individually marked using a metal ornithological ring. In most species, sex identification was possible only for adults, and in some cases, we had difficulties with sexing adult individuals. During the whole season we caught and ringed a total of 245 individuals, including 159 adults and 89 juveniles. Among the individuals that could be sexed, proportions of males and females were roughly equal (95 and 91, respectively). The birds represented 14 species, mostly passerines (Table 1). Before ringing, each bird was visually examined for the presence of ticks during routine handling (standard ornithological right-hand grip; Demongin 2016). Particular attention was paid to the head and bill area as previous studies have revealed that ticks were found only/mostly in these areas (Ciebiera et al. 2019; Zając et al. 2022). Furthermore, ventral areas were also inspected by blowing feathers.

**Table 1:** Numbers of individuals of 14 bird species caught at the Milicz fishponds nature reserve from 17 May to 31 July 2024. We present the total numbers of adults and juveniles of each species. Sex identification was not possible for most juveniles, and 4 adults

Scientific name	Common name	Order	Total no. of adults (male | female)	No. of juveniles	Total no. of individuals
*Acrocephalus arundinaceus*	Great reed warbler	Passeriformes	7 (3 | 3)	2	9
*Acrocephalus schoenobaenus*	Sedge warbler	Passeriformes	10 (5 | 5)	4	14
*Acrocephalus scirpaceus*	Reed warbler	Passeriformes	110 (59 | 48)	19	129
*Carpodacus erythrinus*	Common rosefinch	Passeriformes	1 (0 | 1)	0	1
*Cyanistes caeruleus*	Blue tit	Passeriformes	3 (1 | 1)	5	8
*Schoeniclus schoeniclus*	Reed bunting	Passeriformes	6 (3 | 3)	0	6
*Hirundo rustica*	Barn swallow	Passeriformes	1 (1 | 0)	0	1
*Locustella luscinioides*	Savi’s warbler	Passeriformes	9 (3 | 6)	6	15
*Panurus biarmicus*	Bearded reedling	Passeriformes	2 (1 | 1)	33	35
*Parus major*	Great tit	Passeriformes	1 (0 | 1)	0	1
*Phylloscopus collybita*	Common chiffchaff	Passeriformes	2 (1 | 1)	1	3
*Remiz pendulinus*	Penduline tit	Passeriformes	4 (3 | 1)	3	7
*Sylvia atricapilla*	Eurasian blackcap	Passeriformes	3 (2 | 1)	5	8
*Alcedo atthis*	Common kingfisher	Coraciiformes	0 (0 | 0)	8	8

## Identification and collection of ticks

Ticks were removed from the birds’ bodies with tweezers and placed in an Eppendorf tube containing 70% ethanol solution. The samples were transported to the laboratory where they were identified to species using a stereoscopic microscope, a tick identification key (Estrada-Peña et al. 2017) and molecular methods as described by Gou et al. (2018) based on *COI* mtDNA. DNA was extracted from the ticks using a tissue kit (Tissue DNA Purification Kit, EURx). The universal primers LCO1490 (5′-GGT CAA CAA ATC ATA AAG ATA TTG G-3′) and HCO2198 (5′-TAA ACT TCA GGG TGA CCA AAA AAT CA-3′) were used to amplify the *COI* sequence.

## Sequence and phylogenetic analysis

The resulting nucleotide sequences were subjected to editing using the DNA Baser Sequence Assembly software (Heracle BioSoft S.R.L., Romania) and aligned with reference sequences of *Haemaphysalis* spp. from GenBank using BLAST (Basic Local Alignment Search Tool). Phylogenetic analyses were performed using MEGA X software (Kumar et al. 2018). The tree was constructed using Maximum Likelihood (ML) and bootstrapping was performed using 1000 replicates.

## Results

**Fig. 1:**
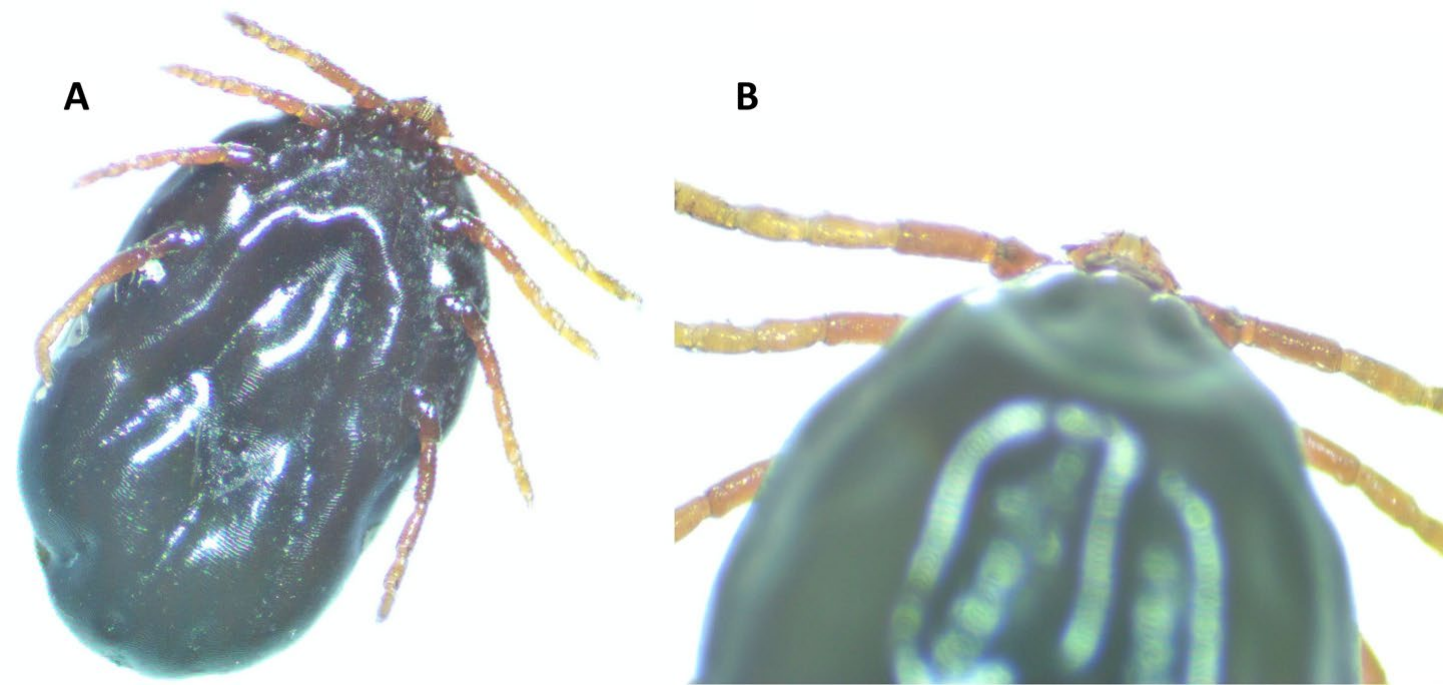
*Haemaphysalis concinna* nymph collected from Great reed warbler (*Acrocephalus arundinaceus*) (**A**, **B**)

**Fig. 2:**
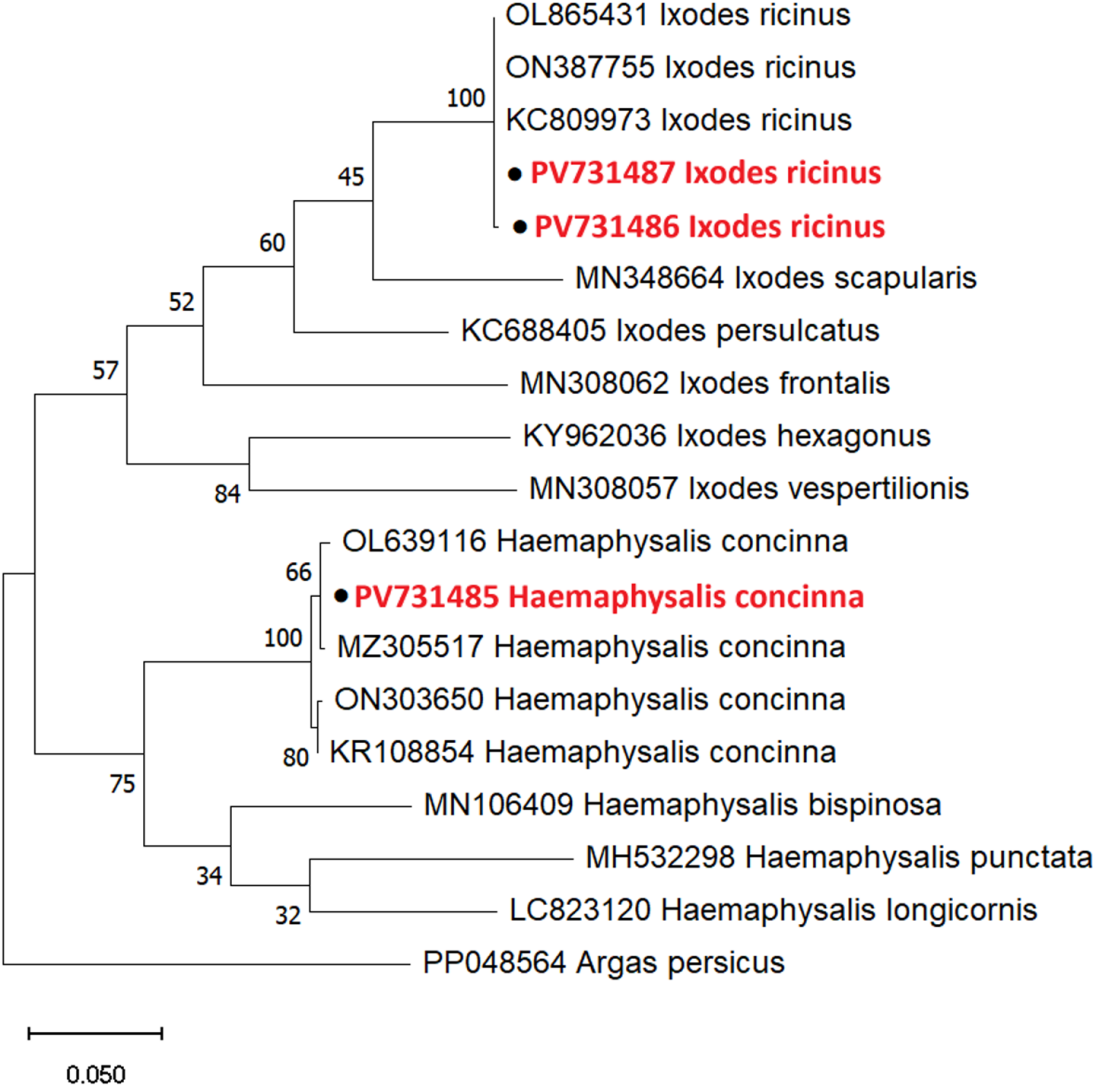
Maximum-likelihood (ML) phylogenetic tree based on partial *COI* sequences of ticks collected from birds. Sequences obtained in this study are marked with solid circles and highlighted in red. Ticks were also morphologically identified as *Ixodes ricinus* and *Haemaphysalis concinna*. Accession numbers from GenBank precede species names. An *Argas* spp. tick sequence was used as an outgroup to root the tree. Numbers represent bootstrap support values based on 1,000 replications (MEGA X software)

Four ticks were collected from three individuals of all captured birds (245 specimens). Overall, the infestation level was 1.22% (95% CI: 0.42%–3.54%), calculated as the proportion of birds infested with at least one tick. The collected ticks were identified as two *Ixodes ricinus* larvae and two *Haemaphysalis concinna* nymphs (GenBank accession number: PV731485 - PV731487). The obtained sequences showed over 98% similarity to *I*. *ricinus* sequences in GenBank for the larval samples identified as *I. ricinus*, and to *H*. *concinna* sequences for the nymphal samples identified as *H. concinna* (Figs. 1 and 2).

*Ixodes ricinus* larvae were collected from sedge warbler *Acrocephalus schoenobaenus*, and Eurasian reed warbler *Acrocephalus scirpaceus* (Table 2). Additionally, two *H. concinna* nymphs were collected from a great reed wabler *Acrocephalus arundinaceus* which was caught at the end of the breeding season (July 10).

**Table 2:** Number of *Ixodes ricinus* larvae and *Haemaphysalis concinna* nymphs collected from different bird species

Bird species	Number of ticks	
	Ixodes ricinus (larvae)	Haemaphysalis concinna (nymphs)
*Acrocephalus schoenobaenus*	1	0
*Acrocephalus scirpaceus*	1	0
*Acrocephalus arundinaceus*	0	2
Total	2	2

## Discussion

Based on previous ornithological and parasitological studies conducted in Poland, it has been shown that both migratory and resident birds are carriers of various hard ticks, including both native and introduced species. So far, among the tick species considered established of Poland’s fauna, the following have been collected from birds: *Ixodes ricinus*, *I. persulcatus*, *I. frontalis*, *I. trianguliceps*, *I. crenulatus*, *I. apronophorus*, *I. arboricola*, *I. eldaricus*, *I. lividus*, *I. caledonicus*,* I. festai*, as well as *Dermacentor reticulatus*,* Haemaphysalis punctate*, and *H. marginatum* (Siuda et al. 2006; Nowak-Chmura and Siuda 2012; Nowak-Chmura et al. 2012; Ciebiera et al. 2019; Zając et al. 2022). Additionally, non-native species of ticks transferred on birds to the territory of Poland are noted: *I. acuminatus* (synonym *I. redikorzevi*), *I. eldaricus*, *I. festai*, and *H. marginatum* (Siuda et al. 2006; Nowak-Chmura et al. 2012; Zając et al. 2022). Birds thus play a key role as passive vectors of parasites over considerable distances, including rare species and non-native species.

In this study, of the 245 birds examined, ticks were found on only three individuals. A total of four ticks were collected: two *Haemaphysalis concinna* nymphs (from *Acrocephalus arundinaceus*) and two *Ixodes ricinus* larvae (from *Acrocephalus schoenobaenus* and *Acrocephalus scirpaceus*). Despite the low level of infestation, this discovery is significant, as there have been no previous reports of *H. concinna* parasitizing birds in Poland. A study by Zając et al. (2022) in the Vistula Valley found that 4.43% of 3,903 birds were infested with ticks, with *I. ricinus* being the dominant species. The researchers also confirmed the significant influence of bird ecology, foraging behaviour and migration distance, on the level of infestation. The highest levels of tick infestation were found in ground-dwelling birds that migrate short and medium distances, such as the common blackbird *Turdus merula* and European robin *Erithacus rubecula*, with 2.73 ticks/bird and 1.58 ticks/bird respectively. A very high infestation of birds with ticks (40.5%) was recorded by Ciebiera et al. (2019) on the Baltic coast, with an average of 2.9 ticks per infected bird, simultaneously showing that the infestation level varies greatly depending on the location and migration period. Although our infestation rate (1.22%) was lower than in the aforementioned studies, it should be emphasized that the majority of the analysed individuals belonged to the group of reed-dwelling birds, which mainly move in the middle and upper layers of vegetation, less often having contact with *I. ricinus* larvae, which actively search for hosts near the ground surface. In contrast, *H. concinna* prefers higher vegetation layers and can infest songbirds in reed beds and bushes more effectively – which is in line with the results obtained by Keve et al. (2024) and is supported by observations from Hornok et al. (2014, 2016).

*Haemaphysalis concinna* has been reported with increasing frequency in Central Europe, including in regions previously considered to be outside its range. In Poland, the first reports of *H. concinna*, originating from a well-documented site, appeared in the 1950 s (Siuda 1993). The subsequent sightings were only noted in the 21 st century, primarily in the western and south-eastern parts of the country (Dwużnik et al. 2019; Kiewra et al. 2019; Zięba et al. 2019; Król et al. 2024). The proximity of these earlier records suggests that the current finding may reflect a pattern of gradual range expansion. However, it is also possible that the growing number of detections, including ours, is at least partly the result of increased awareness and targeted surveillance efforts in recent years. Distinguishing between these scenarios will require coordinated, long-term monitoring across multiple sites and host species. Although our findings of *H. concinna* on *A. arundinaceus* most likely originated from the breeding area, the earlier involvement of birds in the introduction of this species to the Milicz Fishponds Nature Reserve cannot be ruled out. Research by Keve et al. (2024) shows a clear correlation between bird migration and the expansion of this tick’s range in Europe, as well as the presence of *H. concinna* on the *A. scirpaceus*, *A. schoenobaenus* and *A. arundinaceus*. In the context of climate change and the possible expansion of non-native tick species, the role of birds as carriers of parasites and pathogens becomes even more important (Flaisz et al. 2017). Monitoring of ticks in birds, especially those migrating through key ecological corridors, should be continued, taking into account seasonal, habitat, and behavioural variables.

Our discovery of *H. concinna* on birds for the first time in Poland makes these findings particularly important for understanding its ecological dynamics and underscores the need for ongoing surveillance of tick populations and their interactions with migratory birds. As these birds travel long distances, they may facilitate the spread of ticks and associated pathogens across regions, potentially leading to new foci of tick-borne diseases (Keve et al. 2024).

## Conclusion

The first record of *Haemaphysalis concinna* on *Acrocephalus arundinaceus* in Poland expands current knowledge of tick–bird associations in wetland habitats. This finding highlights the importance of avian surveillance for understanding tick biodiversity and suggests the need for further research into the ecological dynamics of bird–tick–pathogen interactions.

## Data Availability

We declare that all data supporting the findings of this study are provided within this manuscript.
